# Mass measurements during lymphocytic leukemia cell polyploidization decouple cell cycle- and cell size-dependent growth

**DOI:** 10.1073/pnas.1922197117

**Published:** 2020-06-24

**Authors:** Luye Mu, Joon Ho Kang, Selim Olcum, Kristofor R. Payer, Nicholas L. Calistri, Robert J. Kimmerling, Scott R. Manalis, Teemu P. Miettinen

**Affiliations:** ^a^Koch Institute for Integrative Cancer Research, Massachusetts Institute of Technology, Cambridge, MA 02139;; ^b^Department of Physics, Massachusetts Institute of Technology, Cambridge, MA 02139;; ^c^Microsystems Technology Laboratories, Massachusetts Institute of Technology, Cambridge, MA 02139;; ^d^Department of Mechanical Engineering, Massachusetts Institute of Technology, Cambridge, MA 02139;; ^e^Department of Biological Engineering, Massachusetts Institute of Technology, Cambridge, MA 02139;; ^f^Medical Research Council Laboratory for Molecular Cell Biology, University College London, London WC1E 6BT, United Kingdom

**Keywords:** mass measurement, cell size, cell growth, cell cycle, transport limitation

## Abstract

Cell size is believed to influence cell growth and metabolism. Consistently, several studies have revealed that large cells have lower mass accumulation rates per unit mass (i.e., growth efficiency) than intermediate-sized cells in the same population. Size-dependent growth is commonly attributed to transport limitations, such as increased diffusion timescales and decreased surface-to-volume ratio. However, separating cell size- and cell cycle-dependent growth is challenging. To address this, we monitored growth efficiency of pseudodiploid mouse lymphocytic leukemia cells during normal proliferation and polyploidization. This was enabled by the development of large-channel suspended microchannel resonators that allow us to monitor buoyant mass of single cells ranging from 40 pg (small pseudodiploid cell) to over 4,000 pg, with a resolution ranging from ∼1% to ∼0.05%. We find that cell growth efficiency increases, plateaus, and then decreases as cell cycle proceeds. This growth behavior repeats with every endomitotic cycle as cells grow into polyploidy. Overall, growth efficiency changes 33% throughout the cell cycle. In contrast, increasing cell mass by over 100-fold during polyploidization did not change growth efficiency, indicating exponential growth. Consistently, growth efficiency remained constant when cell cycle was arrested in G_2_. Thus, cell cycle is a primary determinant of growth efficiency. As growth remains exponential over large size scales, our work finds no evidence for transport limitations that would decrease growth efficiency.

## Significance

Cell size is believed to influence cell growth through limited transport efficiency in larger cells. However, this has not been experimentally investigated due to a lack of noninvasive, high-precision growth quantification methods suitable for measuring large cells. Here, we have engineered large versions of microfluidic mass sensors called suspended microchannel resonators in order to study the growth of single mammalian cells that range 100-fold in mass. Our measurements, which decouple growth effects caused by cell cycle and cell size, revealed that absolute cell size does not impose strict transport or other limitations that would inhibit growth and that cell cycle has a large influence on growth.

The extent to which cell cycle and cell size affect cell growth efficiency (growth rate per unit mass) is not known. Animal cell growth is generally considered exponential (growth rate linearly increases with size resulting in constant growth efficiency) (reviewed in refs. 1–3), and this may be naturally expected, as larger cells have more capacity and machinery to acquire mass. However, studies looking at cultured and proliferating animal cells with high resolution have revealed that the smallest and the largest cells in a population have decreased growth efficiency (4–7). One explanation for the decreased growth in large cells is that, when cells grow beyond a certain size, their growth becomes constrained by transport limitations (2, 8–16). Most notably, larger cells have longer diffusion distances and lower surface-to-volume ratios, both of which could reduce the maximal rate at which they can transfer metabolites and information. Importantly, such transport limitations can exist even when cellular components scale isometrically with cell size. In a developmental setting, growth-influencing transport limitations could have a major impact on cell physiology, possibly explaining why most fast-growing and proliferating cell types are small (<20 µm in diameter) (8–10). Transport limitations are also considered to result in allometric scaling of metabolism, a phenomenon where larger animals display lower metabolic and growth rates (12, 13). However, whether increasing cell size fundamentally imposes transport limitations that result in decreased growth efficiency is not known.

Alternatively, the correlation observed between cell mass and growth efficiency (4–7) could reflect cell cycle-dependent growth, where each specific cell cycle stage has differential growth signaling and metabolism. This growth regulation can be entirely independent of cell size or can be coupled to size-dependent titration/dilution effects, where the concentration of cellular components is lowered as cells grow larger. Such dilution effects often depend on DNA content, and consequently, the dilution effects should be most prominent when cells grow during a cell cycle arrest (3, 16–19). In support of cell cycle-dependent growth, cell cycle regulators are known to influence protein synthesis machinery (20–23), and growth rates in G_1_ have been shown to depend on cell size (7, 24), presumably due to dilution effects. However, as cell cycle stage changes with cell size in most proliferating cell types, measurements must be capable of decoupling cell size and cell cycle effects in order to understand their individual contributions to cell growth.

To quantify the extent of cell size-dependent growth, one would need to examine cells of vastly different sizes. Cultured cells maintain size homeostasis and display little size variability, typically varying over a twofold size range. However, cell size can increase significantly when cells undergo repeated cell cycles in the absence of cell division (polyploidization). Polyploidization and the associated cellular hypertrophy is normal and critical in many tissues during development (8, 14, 25, 26), and also commonly observed in cancers (25, 27). Although the physiological importance of polyploidy is well established, method limitations have prevented high-resolution single-cell measurements of growth in large polyploid cells. Several methods, including quantitative phase microscopy (6, 28), fluorescence exclusion microscopy (7, 29), and suspended microchannel resonators (SMRs) reported thus far (5, 30), are capable of noninvasively quantifying single-cell growth rates of small cells (diameter range from <5 to 15 µm in spherical cells). However, for the large cell sizes observed in polyploid cells, these techniques become imprecise or even infeasible, depending on the method. Here, we expand the analytical range of SMRs by engineering large-channel versions of these devices. We then use the large-channel SMRs together with previously published small-channel SMRs to monitor the growth of vastly different sized single cells and quantify the extent to which cell size and cell cycle influence growth.

## Results and Discussion

**Fig. 1.**
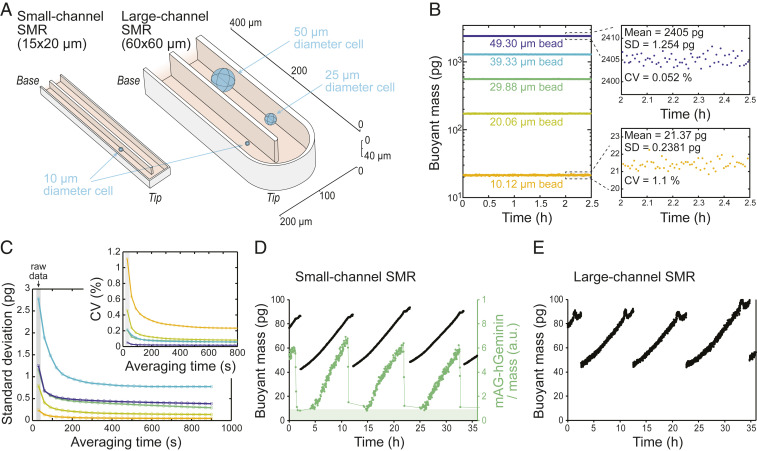
Large-channel SMR enables buoyant mass monitoring across large size ranges with high resolution. (*A*) In-scale schematic of the small- and large-channel SMR cantilevers. The measurement principle of SMRs is to flow a cell through a vibrating cantilever while monitoring a change in the resonant frequency, which is directly proportional to the buoyant mass of the cell. (*B*) Quantification of large-channel SMR resolution based on repeated mass measurements of single polystyrene beads of different sizes (diameters provided by the manufacturer). (*Insets*) Zoom-in views of the 10.12- and 49.30-µm bead data along with measurement mean, SD, and coefficient of variation (CV). (*C*) Large-channel SMR mass measurement resolution as a function of averaging time (moving average filter length reflecting temporal resolution) over multiple measurements for different-sized polystyrene beads. Color coding is the same as in *B*. The measurement interval is ∼30 s, and the first data point under a gray background reflects individual measurements without any averaging. (*Insets*) Measurement resolution as CV. (*D* and *E*) Example mass traces of control L1210 FUCCI cells growing through multiple divisions in small-channel SMR (*D*) and in large-channel SMR (*E*). Data represent individual mass measurements without averaging. At each division, one daughter cell is randomly discarded. The mAG-Geminin signal (green) was only measured in small-channel devices, and its increase indicates G_1_/S transition.

The SMR is a microfluidic mass measurement device where a cell is flown through a vibrating cantilever and the change in the cantilever’s vibration frequency is used to quantify the buoyant mass of the cell. To overcome previous size range limitations, we developed large-channel SMRs, which have a 60 × 60-µm microfluidic channel inside the vibrating cantilever (Fig. 1*A*). These large-channel devices operate in the first vibration mode and utilize a new image-based hydrodynamic trapping approach to repeatedly measure the buoyant mass of a single particle/cell (*SI Appendix*, Fig. S1 and *Materials and Methods*). The image-based hydrodynamic trapping provided additional stability for long-term mass monitoring by allowing us to maintain a cell or a bead in a specified region within the microfluidic channels between measurements. Using polystyrene beads, we quantified each large-channel SMR mass measurement to have a resolution (standard deviation; SD) ranging between 0.24 and 1.25 pg for particles ranging from 10 µm (21.37 pg) to 50 µm (2405 pg) in diameter, respectively (Fig. 1*B*; see *SI Appendix*, Fig. S1, and *Materials and Methods* for full details). This corresponds to a measurement coefficient of variation range from 1.1 to 0.05%, respectively. When monitoring single-cell growth, we were able to acquire mass measurement every ∼30 s without affecting cell viability (see comparisons of cell growth in small and large-channel SMRs below), allowing us to average multiple mass measurements when monitoring mass changes that take place over longer time periods (Fig. 1*C*).

To validate that the large-channel SMRs provide data comparable to previous 15 × 20-µm SMRs (from here on referred to as small-channel SMRs), we measured single-cell buoyant mass accumulation rate (from here on referred to as growth rate) of L1210 cells (suspension-grown pseudodiploid mouse lymphocytic leukemia cell line) expressing the mAG-hGeminin cell cycle reporter (FUCCI). These cells display an adder-like cell size homeostasis mechanism, where the mass added in each cell cycle is independent of cell size at birth (*SI Appendix*, Fig. S2 *A*–*C*) (7). In addition, cell size variability decreased from birth to G_1_/S transition (*SI Appendix*, Fig. S2*D*) (5), and cell cycle duration had a weak negative correlation with cell size at birth (*SI Appendix*, Fig. S2*E*). It is also worth pointing out that these cells have a short G_1_ and overall cell cycle duration (∼3.5 and ∼10.5 h, respectively). We have previously shown that cell cycle durations in small-channel SMRs are identical to those in bulk culture (5, 31), and we did not observe differences between small- and large-channel SMRs in cell growth rates, cell cycle durations, or cell division symmetries (*SI Appendix*, Fig. S3). It is known that growth rate, cell density, and cell stiffness display dynamic changes in mitosis (20, 31, 32). As these changes are unlikely to reflect cell size-dependent effects, we have excluded mitosis from all analyses. While the small-channel SMR has better measurement resolution than the large-channel SMR when measuring normal-sized L1210 cells (SDs of 0.026 and 0.24 pg for a 10-µm diameter bead of 21.37 pg on small- and large-channel SMR, respectively) (20), the large-channel SMR increases the maximum spherical cell volume that can be measured 64-fold. Importantly, the large-channel SMR is also able to monitor growth of a single cell over multiple cell cycles (randomly following one of the daughter cells at each division; Fig. 1 *D* and *E*), which has previously been achieved by only a few cell size measurement methods (1).

**Fig. 2.**
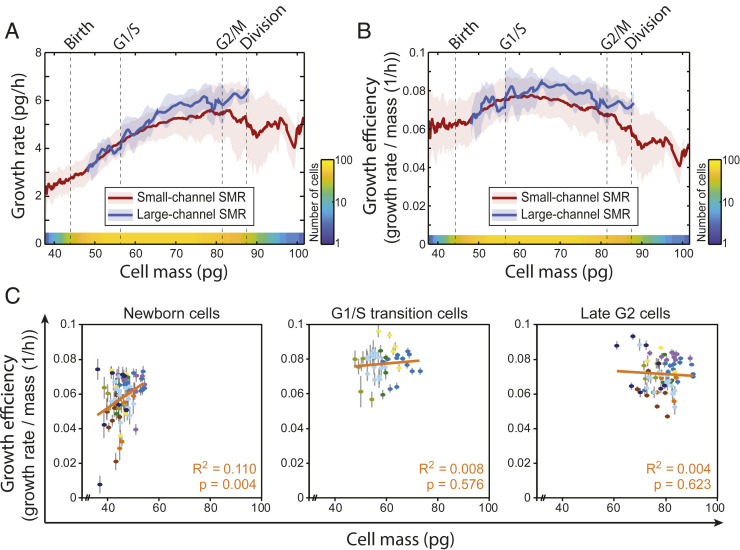
Growth efficiency of unperturbed cells correlates poorly with cell size within specific cell cycle stages. (*A* and *B*) The growth rate (*A*) and growth efficiency (*B*) of L1210 FUCCI cells as a function of mass as obtained using small-channel SMR (red traces; number of cells for each size analyzed is indicated with a color gradient at the bottom; *n* = 9 independent experiments, *n* = 64 cells) and large-channel SMR (blue traces; *n* = 2 independent experiments, *n* = 9 cells). The line and shaded area indicate mean ± SD. Average newborn size (Birth), G_1_/S transition size, mitotic entry size (G_2_/M), and division size are indicated with dashed vertical lines. (*C*) Correlations between L1210 FUCCI cell mass and growth efficiency at the beginning of G_1_ (*n* = 9 independent experiments; *n* = 72 cells), at G_1_/S transition (*n* = 5 independent experiments; *n* = 41 cells), and at the end of G_2_ (*n* = 9 independent experiments; *n* = 72 cells). The color indicates each independent experiment. Each cell (dot) is plotted with error bars (measurement error as SD). Linear fits, Pearson correlations (*R*^2^), and *P* values for the correlations (two-tailed test of significance) are shown in orange.

We first studied the size dependency of cell growth by monitoring unperturbed L1210 cells using the small-channel SMRs. Our data revealed that when cells are small (G_1_ and early S-stage cells), growth rate increases with size (as cell cycle proceeds), but then plateaus in larger cells (late S-stage and G_2_ cells) (Fig. 2*A* and Dataset S1). Consequently, the intermediate-sized cells (S-stage cells) displayed the highest growth efficiency (Fig. 2*B*). Large-channel SMRs provided similar data. These results are consistent with previous findings (4–7) that cell size and/or cell cycle have a major effect on cell growth efficiency. Notably, the nonlinear growth behavior was also clear when examining individual cells (*SI Appendix*, Fig. S4). Thus, these results show that the mode of growth cannot be simplified as exponential or even biphasic, but instead L1210 cells gradually change their growth rates throughout the cell cycle.

We next analyzed how growth efficiency scales with time since birth or with time since G_1_/S transition. We found that L1210 cells displayed maximum growth efficiency ∼4.5 h after birth and 1 h after G_1_/S transition (*SI Appendix*, Fig. S5 *A*–*D*). We then examined whether growth efficiency is maximized simply after a given time from cell division (*SI Appendix*, Fig. S5 *E* and *F*). We correlated the total cell cycle length with the timing of maximal growth efficiency and found a positive correlation, indicating that the maximum growth efficiency is not achieved after a fixed time following birth. This suggests that cells maximize their growth efficiency, on average, in early S phase, either due to cell size- or cell cycle-dependent growth.

We then examined how cell size influences growth efficiency independently of the cell cycle stage. We first studied untreated cells, where we measured growth efficiency specifically in particular cell cycle stages (i.e., newborn G_1_, G_1_/S transition, and late G_2_). This revealed little to no correlation between growth efficiency and cell mass within each cell cycle state (Fig. 2*C*). Since the biological variation is much larger than our measurement noise (20), the lack of correlation is unlikely to be due to lack a of measurement precision. Thus, these results suggest that cell size does not have a major influence on L1210 cell growth efficiency when examining changes over small size ranges.

**Fig. 3.**
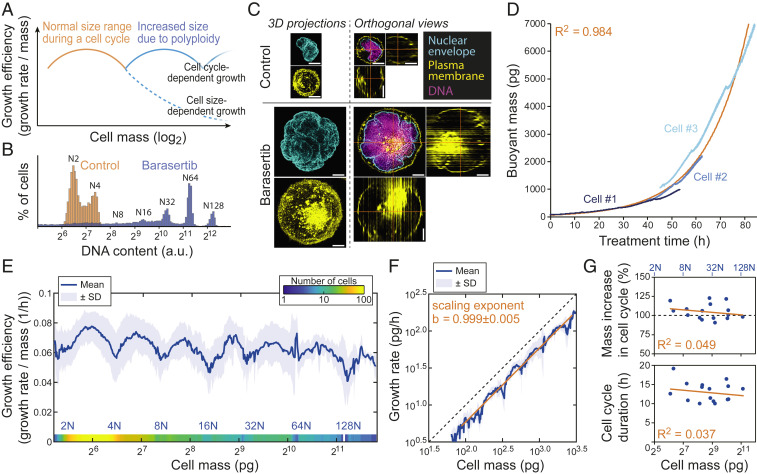
Monitoring growth of polyploid cells over a 100-fold size range reveals that growth is not size limited. (*A*) Hypothesis of cell growth regulation as cells increase size during endomitotic cycles. In the size range observed during a normal cell cycle (orange), cells display nonlinear size scaling of growth efficiency. When cells grow into polyploidy, cell size-dependent (dashed blue line) and cell cycle-dependent (solid blue line) growth should result in different growth behaviors. (*B*) Representative DNA histograms of control (orange) and 80-h 50 nM Barasertib-treated (blue) L1210 FUCCI cells (*n* = 3 independent cultures). (*C*) Representative morphologies of control (*Top*) and 50 nM Barasertib-treated (*Bottom*) L1210 FUCCI cells based on nuclear envelope (cyan), plasma membrane (yellow), and DNA (magenta) staining (*n* = 2 independent experiments each with >10 fields of view). Three-dimensional projections (*Left*) and single *z* slices with orthogonal views (*Right*) are displayed. (Scale bars: 5 µm.) (*D*) Three example buoyant mass traces from 50 nM Barasertib-treated L1210 FUCCI cells obtained using the large-channel SMR. Exponential fit to the three examples and Pearson correlation (*R*^2^) are displayed in orange. (*E*) The combined growth efficiency of control, 50 nM Barasertib, and 10 µM H-1152–treated L1210 FUCCI cells across a large mass range as measured with both small- and large-channel SMRs (*n* = 76 independent experiments across all conditions, number of cells is indicated with color gradient at the bottom). Estimated ploidy level is displayed on bottom in blue. (*F*) Growth rate as a function of mass on a log_10_–log_10_ scale when analyzing only 50 nM Barasertib-treated L1210 FUCCI cells measured with the large-channel SMR (*n* = 31 independent experiments). Linear fit and scaling exponent *b* (mean ± SEM) are displayed in orange. Perfect isometric scaling (*b* = 1) is illustrated with dashed black line. (*G*) Correlations between cell mass at the beginning of each cell cycle and percent mass increase during each cell cycle (*Top*) and cell cycle duration (*Bottom*). The data represent 50 nM Barasertib-treated L1210 FUCCI cells measured with the large-channel SMR (*n* = 11 independent experiments, *n* = 16 endomitotic cycles). The dashed black line at *Top* represents a perfect mass doubling in each endomitotic cycle. Approximate ploidy level at the start of each cell cycle (blue); linear fits (orange) and Pearson correlations (*R*^2^) are displayed.

To examine size-dependent growth over a much larger size range, we utilized polyploid model systems. If the declining growth efficiency observed in the largest unperturbed cells (Fig. 2*B*) is due to transport limitations caused by increases in cell size, then increasing size further by induction of polyploidy should result in further declining growth rates (Fig. 3*A*). However, if the nonlinear correlation between cell size and growth efficiency observed in control cells reflects cell cycle-dependent growth, then the oscillating growth efficiency should repeat with every successive cell cycle in the polyploid cells. To test our hypothesis, we induced polyploidy in L1210 cells using 50 nM Barasertib (also known as AZD1152-HQPA), a selective inhibitor of Aurora B, which is critical for cytokinesis (33, 34). This resulted in several endomitotic cycles where ploidy increased from 2N up to 128N (Fig. 3*B*) with corresponding increases in cellular hypertrophy (Fig. 3 *C* and *D*), suggesting that DNA-to-cell size ratio remained comparable to control cells. Importantly, the cells remained spherical with a single, multilobed nucleus (Fig. 3*C*). Prolonged drug treatments also resulted in cell death, which manifested in mass measurements as sudden transition to zero or negative growth (*SI Appendix*, Fig. S6 *A*–*C*). These data were excluded from our analysis (*Materials and Methods*).

When examining growth over larger size scales using the polyploid cells, mass increased exponentially over time (Fig. 3*D* and *SI Appendix*, Fig. S6*D*). Remarkably, the nonlinear growth efficiency behavior that was observed in control cells (Fig. 2*B*) repeated in every successive cell cycle during polyploidization despite large increases in cell size (Fig. 3*E*). This oscillating growth pattern within each endomitotic cycle was also observable in individual cells (*SI Appendix*, Fig. S7 *A*–*C*). This nonlinear growth behavior cannot be explained by the DNA-to-cell size ratio alone, as growth efficiency decreased toward the end of each cell cycle but started to increase immediately following endomitosis before the subsequent S stage. Furthermore, the low growth efficiency in newborn G_1_ cells (Fig. 2 *B* and *C*) does not result from their small size, as polyploid G_1_ cells, which are considerably larger, display similarly low growth efficiency (Fig. 3*E*). To validate that the observed growth behavior cannot be attributed to drug-specific effects, we induced polyploidy using an alternative cytokinesis inhibitor, 10 µM H-1152, which targets the Rho-kinase (ROCK) (35). This resulted in similar growth behavior as Aurora B inhibition (*SI Appendix*, Fig. S7*A*).

Overall, we quantified growth efficiency for L1210 cells over a ∼100-fold mass range spanning from 40 to 4,000 pg (Dataset S1). In spherical L1210 cells, this corresponds to a diameter range from <7 to >32 µm resulting in estimated 4.5-fold reduction in surface-to-volume scaling. This size range covers most proliferating cell types in the human body. Unlike cells in vivo, cultured cells are constantly selected for the highest growth rate, allowing us to assume that the measured growth rates reflect maximal growth rates possible for the cells. Size scaling typically follows a power law *Y* = *aM*^*b*^, where *Y* is the observable biological feature, *a* is a normalization constant, *M* is the mass of the organisms (or a cell), and *b* is the scaling exponent which typically has values close to 3/4 when studying metabolic rate (12, 13). We observed a minor decrease in growth efficiency in the largest cells when plotting data obtained across multiple measurement systems and conditions (Fig. 3*E*). We therefore quantified size-dependent growth and the allometric scaling exponent from our growth rate data using only Barasertib-treated cells monitored with the large-channel SMR (*SI Appendix*, Fig. S7*D*) After accounting for the oscillating cell cycle-dependent growth (*Materials and Methods*), the L1210 cell growth rates display an isometric scaling exponent of 0.999 ± 0.005 (mean ± SEM) (Fig. 3*F*), consistent with previous predictions for cells in vitro (36). This corresponds to each doubling of cell mass changing growth efficiency by −0.1 ± 0.3% (mean ± SEM), indicative of exponential growth over a large cell size and ploidy range.

**Fig. 4.**
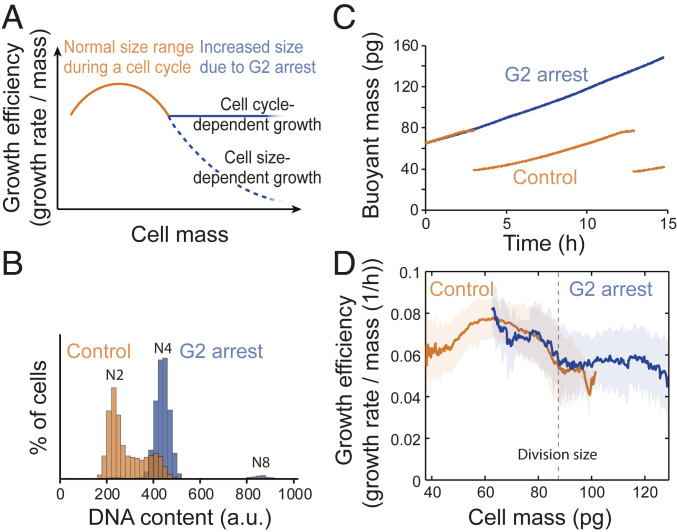
G_2_ cell cycle arrest results in steady growth efficiency. (*A*) Hypothesis of cell growth regulation as cells increase size during a G_2_ cell cycle arrest. In the size range observed during a normal cell cycle (orange), cells display nonlinear size scaling of growth efficiencies. When cells are arrested in G_2_, cell size-dependent (dashed blue line) and cell cycle-dependent (solid blue line) growth should result in different growth behaviors. (*B*) Representative DNA histograms of control (orange) and 24-h 2 µM RO-3306–treated (blue) L1210 cells (*n* = 3 independent cultures). RO-3306 results in a G_2_ arrest, and most cells do not undergo endoreplication cycles. (*C*) Example buoyant mass trace of a control (orange) and 2 µM RO-3306–treated (blue) L1210 cell obtained using small-channel SMR. (*D*) The growth efficiency of control (orange; *n* = 9 independent experiments, *n* = 64 cells) and 2 µM RO-3306–treated (blue; *n* = 12 independent experiments, *n* = 12 cells) L1210 cells. All experiments with RO-3306 lasted under 24 h to avoid cell death. The solid lines and shaded areas indicate mean ± SD. The dashed vertical line indicates the typical division size of control cells.

In contrast to cell size, cell cycle displays a strong influence over cell growth efficiency. To validate that cell cycle progression causes the oscillating growth behavior within each cell cycle, we arrested L1210 cells to G_2_ stage with 2 µM RO-3306, an inhibitor of cyclin-dependent kinase 1 (CDK1) (37) (Fig. 4 *A* and *B*). Prolonged RO-3306 treatment resulted in cell death, and to avoid this toxicity, we only analyzed growth for the first 40-pg increase (corresponding to a typical mass increase during an unperturbed cell cycle) from the normal mitotic size. This revealed that the decrease in growth efficiency that was observed in large control cells stopped as cells were arrested in G_2_ and the growth efficiency remained constant for G_2_ arrested cells even as their sizes increased (Fig. 4 *C* and *D* and *SI Appendix*, Fig. S8). Thus, as suggested by previous work in budding yeast (38), our results show that cell cycle has a major influence on mammalian cell growth efficiency. We quantified this cell cycle-dependent growth to be 33 ± 4% (mean ± SEM) of the average growth efficiency in untreated L1210 cells. In addition, the steady growth efficiency observed in G_2_ arrested cells validates that increasing cell size does not automatically result in decreasing growth efficiency even in a model where DNA content does not scale with cell size. Furthermore, these results suggest that G_2_ growth efficiency is not regulated by dilution of components produced in earlier cell cycle stages.

Finally, using the polyploidy cell data collected by the large-channel SMR, we also analyzed how cell size increase and cell cycle duration scale with cellular hypertrophy and the associated polyploidy. This revealed that with each successive endomitotic cycle, the L1210 cells approximately doubled their size independently of the cell size at the start of that cell cycle (Fig. 3 *G*, *Top*). Cell cycle duration also remained constant regardless of cell size (Fig. 3 *G*, *Bottom*). This suggests that massive cellular hypertrophy and the associated polyploidy do not interfere with the mechanism(s) ensuring that cells double their size during each cell cycle.

In conclusion, increasing cell size does not impose strict transport limitations that would lower growth efficiency in cultured mammalian cells. This conclusion was reached when observing freely proliferating cells in specific cell cycle stages (Fig. 2*C*), when examining cells across a vast size range following chemically-induced polyploidy (Fig. 3*F* and *SI Appendix*, Fig. S7*D*), and when examining G_2_ arrested cells (Fig. 4*D*). Cells may be able to compensate for the increased intracellular distances and decreased surface-to-volume ratio, for example by up-regulating the expression of active transporters. Alternatively, the transport limitations may only influence growth under very specific conditions, for example when specific nutrients are in low abundance or when cell size increases above the range normally observed in proliferating mammalian cells. In addition, our observations in freely proliferating and in G_2_ arrested cells suggest that the growth effects we observe are due to size, not ploidy, at least over small size ranges. However, increased ploidy, which typically correlates with cell size (39), may be essential for larger size increases, as suggested by work in budding yeast (17).

Our results also show that the previously observed correlation between cell size and growth efficiency in proliferating cultures (4–6) can be explained by cell cycle effects, which have a large influence over cell growth efficiency. While our conclusions rely on data from a single suspension-grown leukemia cell line, L1210, the growth profiles of L1210s and various adherent cells have been shown to be similar (6), suggesting similar cell cycle-dependent growth regulation across multiple cell types. Notably, our results do not exclude growth regulation by dilution effects in G_1_ and S stages of the cell cycle, nor do our results exclude dilution/concentration effects regulating cell cycle progression or cell metabolism (3, 16–19, 40, 41). In fact, size-dependent dilution effects are likely to be responsible for cell size homeostasis, as our data show that cell size homeostasis is not achieved simply by coupling cell growth efficiency to the absolute size of cells.

Methodologically, we anticipate that the large-channel SMRs will have important uses outside this study. The ability to monitor the mass of unlabeled large samples will enable growth (5, 20), drug response (42), and nutrient uptake (43) studies in various models. These include extremely large single cells such as adipocytes or megakaryocytes, as well as individual organoids or tumor spheroids, where adherent cell mass accumulation can now be monitored in a preserved 3D microenvironment.

## Materials and Methods

For detailed materials and methods, please refer to *SI Appendix*.

### SMR Setup, Operation, and Measurement Resolution.

Small-channel SMRs were built and operated as detailed in refs. 20 and 31. Large-channel SMRs were fabricated at Massachusetts Institute of Technology, driven using a piezo-ceramic placed underneath the SMR chip. Its vibration frequency was measured using an optical lever technique (42). The large-channel SMRs were operated in the first flexural bending mode with a typical resonance frequency around 420 kHz. The image-based hydrodynamic trapping is detailed in *SI Appendix*, Fig. S1*B*. In all experiments, cells were measured approximately every 30 to 60 s. The measurement precision of large-channel SMRs was determined by repeatedly measuring a single polystyrene bead (Duke standard 4000 series; Fisher Scientific). When measuring cell growth, cells inside the SMR were exposed to culture conditions detailed below.

### Cell Culture and Chemical Treatments.

Experiments were carried out using mAG-hGeminin–expressing L1210 FUCCI cell line, which was generated in a previous study (5) and originated from ATCC (catalog no. CCL-219). However, the RO-3306 treatment experiments were carried out using the parental L1210 cells, as the FUCCI cells displayed higher RO-3306 toxicity than the parental cells. Cells were grown in RPMI media (containing 11 mM glucose, 2 mM l-glutamine, 10% FBS, 1 mM sodium pyruvate, 20 mM Hepes, and antibiotic/antimycotic) at 37 °C in 5% CO_2_ and 21% O_2_ atmosphere. Cells tested negative for mycoplasma. Barasertib (also known as AZD1152-HQPA; Cayman Chemical; catalog no. 11602), H-1152 (Sigma-Aldrich; catalog no. 555550), and RO-3306 (Cayman Chemical; catalog no. 15149) were dissolved in DMSO. The chemical concentrations used were selected based on cell cycle phenotypes observed in control experiments.

### Data Analysis.

SMR frequency data were analyzed using custom MATLAB codes and converted to buoyant mass using sensitivity factors obtained from polystyrene bead measurements (20, 31) (*SI Appendix*, *Materials and Methods*). For all samples, except for RO-3306–treated cells, we only analyzed mass traces that contained one or more mitosis, but mitotic regions (from G_2_/M transition to 15 min after metaphase/anaphase transition) were excluded from our growth analyses. If cells died while trapped within the SMR, we excluded the part of the data where mass accumulation was zero or negative (see *SI Appendix*, Fig. S6*C* for examples). When analyzing control cells using the small-channel SMR, we always monitored the cells for multiple cell cycles to verify that our analysis focused on actively growing and proliferating cells. The quantification of cell size-dependent growth was carried out using Barasertib-treated L1210 cell data from the large-channel SMRs exclusively. The cell size-dependent growth was determined based on the slope of a line fitted to the growth efficiency data spanning five cell cycles (*SI Appendix*, Fig. S7D). The cell cycle-dependent growth efficiency was determined by comparing the typical maximal and minimal growth efficiency observed within an unperturbed cell cycle.

### Data AvailabilityStatement.

All data are included in the manuscript and Dataset S1.

## Supplementary Material

Supplementary File

Supplementary File
