# Supplementary material for: Mass measurements during lymphocytic leukemia cell polyploidization decouple cell cycle- and cell size-dependent growth
Source: Proc Natl Acad Sci U S A. 2020 Jun 24;117(27):15659–65. doi: 10.1073/pnas.1922197117 (PMC7355023; doi:10.1073/pnas.1922197117)
Supplement: Supplementary File [file pnas.1922197117.sapp.pdf]

PNAS
www.pnas.org

Supplementary Information for

Mass measurements during lymphocytic leukemia cell polyploidization decouple cell cycle and cell size dependent growth

Luye Mu, Joon Ho Kang, Selim Olcum, Kristofor R. Payer, Nicholas L. Calistri, Robert J. Kimmerling, Scott R. Manalis and Teemu P. Miettinen

Scott R. Manalis
Email: srm@mit.edu

Teemu P. Miettinen
Email: teemu@mit.edu

This PDF file includes:

SI Materials and Methods
Figures S1 to S8
Legends for Figures S1 to S8
Legend for Dataset S1
SI References

Other supplementary materials for this manuscript include the following:

Dataset S1

www.pnas.org/cgi/doi/10.1073/pnas.1922197117

SI Materials and Methods

Small-channel SMR details and operation

Small-channel SMRs were built and operated as detailed in (1, 2). Briefly, the silicon chips that contain the vibrating cantilever were fabricated by CEA-LETI (Grenoble, France). The length of the cantilever used in this study was 350 μm and the buried channel cross section size was 20 × 15 μm (width × height). The small-channel SMR was driven using a piezo-ceramic placed under the device and operated in the second flexural bending mode, with a typical resonance frequency of approximately 1.1 MHz. Piezoresistors embedded at the base of the cantilever were used to monitor the resonance frequency. The cantilever was actuated using a digital control platform in a direct feedback mode with a typical data rate of 3000 Hz. The SMR device was mounted on a metal clamp with water circulating inside the clamp. The water, and consequently the SMR, was maintained at 37°C by a temperature-controlled water bath (Julabo). Each input port to the SMR was pressurized with 5% CO_2, 21% O_2-containing gas (Airgas) to maintain a stable pH (3). The pressure in each input, as well as cantilever actuation, were controlled using a custom-made software (LabVIEW 2016) in order to operate the SMR and control the flow of fluids within the SMR. A typical flow rate within the SMR channels was 2 nL/s, resulting in approximately 200-300 ms transit time through the small-channel SMR cantilever (2). The trapping of a cell within the SMR was obtained by an automated system that adjusts pressures to momentarily stop fluid flow following a resonant frequency change (cell transiting through the cantilever). Approximately 50 s after the fluid flow was stopped, the fluid flow direction was automatically reversed and the measurement of the same cell was repeated. This resulted in a typical measurement interval of 1 min. In addition, the small-channel SMR is equipped with a fluorescence detection setup detailed in (1, 4). This consists of a modular Nikon microscope equipped with a Nikon LU plan ELWD 50x/0.55 objective and an 8 mm Voltage Output Type photomultiplier tube (Edmund Optics). Illumination using a Lumencor Spectra X light engine was only activated for 400 ms following cell transit through the cantilever, during which the cell transited through the illumination area and fluorescence signal from the FUCCI cell cycle reporter was detected. We carried out experiments with and without excitation of the FUCCI sensor and did not observe a difference in growth rates. All SMR experiments were carried out by growing the cell within the SMR using the same culture media and chemical perturbations that are detailed below and in figure legends.

Large-channel SMR fabrication and physical dimensions

Large-channel SMR chips were fabricated on six-inch silicon wafers using a three-level process at the Microsystems Technology Laboratories at MIT. The base device wafer is a double silicon-on-insulator (SOI) substrate (Ultrasil), consisting of a 60 μm silicon layer, a 100 nm buried oxide layer, another 5 μm silicon layer, a 1 um buried oxide layer, and a 500 μm thick silicon handle. Microfluidic channels are etched 60 μm deep in the top silicon layer using deep reactive ion etching (DRIE), with the first buried oxide as etch stop. The base device wafer is then fusion bonded to a device lid wafer, which consists of a standard SOI wafer with 5 μm thick top silicon layer (Ultrasil). The resulting stack is mechanically thinned from the back of the device lid wafer, which yields a structure that contains buried microfluidic channels with 5 μm caps on the top and bottom. To allow fluidic access to the buried channel, an etch through the lid opens the microfluidic channel to the wafer surface. Subsequently, a 70 μm DRIE step defines the cantilever by etching through all of the SOI layers down to the base wafer handle. Microfluidic bypass channels are wet etched into a Pyrex wafer (Bullen) with hydrofluoric acid. Inlet and outlet ports are drilled through the Pyrex wafer with an ultrasonic drilling process. The Pyrex microfluidic wafer is anodically bonded to the top of the device stack. The cantilever is released by DRIE through the silicon from the backside, followed by a brief

reactive ion etch to remove the buried oxide. Unlike the small-channel SMRs, these devices were fabricated without piezoresistive readout elements, and the cantilever cavity was left open to atmosphere. See (*SI Appendix*, Fig. S1A) for an image of an example large-channel device. All large-channel experiments were carried out on devices with buried channel cross section size of 60 × 60 µm, and cantilever length of 407 µm.

Large-channel SMR operation

The large-channel SMRs were driven using a piezo-ceramic placed underneath the SMR chip. Due to spurious resonances close to the second mode frequency, we operated the devices in the first flexural bending mode. The typical resonance frequency was approximately 420 kHz. In order to overcome position-dependent error at the tip associated with first mode operation, we used flow focusing to confine the transit path of the particle (3). The SMR vibration frequency was measured using an optical lever technique similar to work previously reported (5). The resulting signal is fed into a phase-locked loop at a data rate of 4000 Hz, with a loop bandwidth of 100 Hz (5, 6). Other aspects of the setup, including pressure and temperature control are similar to that of the small-channel SMR. Pressures were controlled to achieve 200-300 ms transit times through the cantilever. In order to improve trap stability, an image-based trapping technique was used to repeatedly measure the cell in the large-channel device. The image-based detection and fluidic control is achieved using a custom-made software in Labview (see *SI Appendix*, Fig. S1B for operating principle). Cells were measured approximately every 30-60 s in each experiment. We reduced the engineering complexity of the optics by omitting fluorescence measurement capabilities in the large-channel SMRs.

All SMR experiments were carried out by growing the cell within the SMR using the same culture media and chemical perturbations that are detailed below and in figure legends. Before each experiment, the device was cleaned with 10% bleach followed by a thorough rinse in deionized water. The channel walls are passivated with 1 mg/mL PLL-g-PEG in deionized water for 10 min before flushing the device with measurement media.

Large-channel SMR measurement error and linearity

Large-channel SMR calibration was performed using polystyrene beads of known sizes (Duke standard 4000 series purchased from Fisher Scientific) to derive mass sensitivity factors for each device (*SI Appendix*, Figs. S1C-E). The measured frequency changes were scaled by the sensitivity factor to yield buoyant mass. Sensitivity factor was calculated using different sized beads, but for consistency all cell data was scaled by calibration factors obtained using 30 µm beads measured in PBS at 37°C. Data in (Figs. 1B,C, *SI Appendix*, Figs. S1C-E) were obtained without heating.

The measurement resolution of the large-channel SMRs was determined by repeatedly measuring a single polystyrene bead and calculating the standard deviation of the repeated measurements (Fig. 1B). This reflects error on a single mass measurement (measurement interval is approximately 30 s). To assess how our measurement resolution can be improved to capture mass changes that take place over longer time scales, we analyzed the repeated bead measurements by applying moving average filters of various lengths on to the data (Fig. 1C). Overall, the large-channel SMR measurement noise derives from two main sources. First, the SMRs have background frequency noise (~30-50 mHz). Second, as the cell or a particle flows through the cantilever and turns around at the tip of the cantilever, the flow path (i.e. how close to the tip of the cantilever the cell flows) will influence our measurement. To minimize the measurement-to-measurement variability in the flow path, we balance fluid flows so that the cells is maintained close to the outer edge of the cantilever (flow focusing). However, the flow focusing can vary from day-to-day and from cell-to-cell. The large-channel SMR measurement resolution reported in this study

reflects error caused by both flow path and frequency noise. Mass resolution of smallest and largest particles are dominated by frequency noise, whereas intermediate sized particles are dominated by flow focusing noise.

We also evaluated the linearity of the large-channel SMR mass measurements by calculating the mass sensitivity factor for vastly different sized polystyrene beads. Assuming that the different sized polystyrene bead standards are fully comparable in density and shape, these measurements suggested a small deviation from fully linear scaling between buoyant mass and frequency change (*SI Appendix*, Fig. S1E). This small non-linearity could also reflect day-to-day variability in the SMR measurements originating from small changes in flow focusing, room temperature and humidity, solution density etc., or acoustic effects that are specific to hard particles (2). Importantly, the metric of growth efficiency is insensitive to gradual changes in device calibration, as we calculate growth efficiency using small mass intervals accumulated over 15 min or 60 min periods (see *SI Appendix SMR data analysis and statistics*). Although the mass values are affected by calibration, we see approximate doubling of cell mass with each mitosis (Fig. 3G), indicating that this variability is either bead-specific, or the effects are averaged out for cell measurements conducted over multiple days.

Cell culture and chemical treatments

Experiments were carried out using mAG-hGeminin expressing L1210 FUCCI cell line, which was generated in a previous study (3) and originated from ATCC (Cat#CCL-219). All experiments were carried out using RPMI media that contained 11 mM glucose, 2 mM L-glutamine, 10% FBS (Sigma-Aldrich), 1 mM sodium pyruvate, 20 mM HEPES and antibiotic/antimycotic. All media components were obtained from Thermo Fisher Scientific unless otherwise stated. Cells were grown at 37°C in 5% CO_2 and 21% O_2 atmosphere. Cells tested negative for mycoplasma.

Barasertib (also known as AZD1152-HQPA) was obtained from Cayman Chemical (Cat#11602), H-1152 was obtained from Sigma-Aldrich (Cat#555550) and RO-3306 was obtained from Cayman Chemical (Cat#15149). We only used fresh stocks (dissolved for <2 weeks) of RO-3306 due to previous observations regarding drug efficacy after longer storage (1). FUCCI expressing L1210 cells displayed higher toxicity following RO-3306 treatment and, consequently, the RO-3306 experiments were carried out using the parental L1210 cell line. All chemicals were dissolved in DMSO and final DMSO concentration was maintained below 0.25%. We did not observe differences in control cell growth with this DMSO concentration. The chemical concentrations used were selected based on cell cycle phenotypes observed in control experiments.

Microscopy

Microscopy samples were prepared on poly-L-lysine (Sigma-Aldrich, Cat#P8920) coated microscopy slides. L1210 cells were first treated with indicated chemicals and grown under normal culture conditions until one hour prior to fixation when the cells were moved to the microscopy slides. Cells were then washed twice with PBS, fixed with 4% paraformaldehyde for 10 min at RT, washed once with PBS and permeabilized with 0.25% Triton X-1000 in PBS for 10 min at RT. Following one PBS wash the cells were incubated with 5% BSA in PBS for 30 min at RT. Nuclear envelope labeling was carried out o/n at 4°C with Lamin A/C mouse monoclonal antibody conjugated to AlexaFluor 488 (Cell Signaling Technology, Cat#8617) using a 1:50 antibody dilution in PBS supplemented with 5% BSA. The following day, the cells were washed twice with PBS, and membrane labeling was carried out o/n at 4°C with Wheat germ agglutinin conjugated to AlexaFluor 594 (Thermo Fisher Scientific, Cat#W11262) using a 2.5 µg/ml dilution in PBS, as shown before (7). The following day, the cells were washed twice with PBS, and DNA labeling was carried out for 1h at RT with 1:2000

dilution of NuclearMask Blue (Thermo Fisher Scientific, Cat#H10325) in PBS. Following two washes with PBS, the cells were mounted using Vectashield mounting medium (Vector Laboratories).

Cells were imaged at RT using DeltaVision wide-field deconvolution microscope with standard filters (DAPI, FITC, Alexa594), a 100x objective and immersion oil with refractive index of 1.516 (Cargille Laboratories). Z-slices were collected with 0.3 μm spacing so that the whole cell volume was covered. Image deconvolution was carried out automatically by the SoftWoRx 7.0.0 software.

Only linear transformations to brightness and contrast were carried out and all images were processed in identical manner. Orthogonal views of representative cells and z-slices were prepared using the 'orthogonal views' function in ImageJ. Location from which the orthogonal view is made from is shown using an orange crosshair in (Fig. 3C). The 3-dimensional z-projections were made using maximum intensity projection function in ImageJ.

The growth and morphology of live L1210 cells was monitored using the IncuCyte live cell analysis imaging system by Sartorius. The images displayed in (*SI Appendix*, Fig. S6B) are an overlay of the phase image and green fluorescence signal image (FUCCI cell cycle sensor) obtained directly from the software without manual corrections. All experiments with Incucyte were carried out by plating cells on a 96-well plate with chemical perturbations in the media. Imaging was performed once every hour using 20X objective and 300 ms exposure time for the FITC channel.

Cell cycle analysis and ploidy estimation

DNA content was analyzed similarly as before (7). Following indicated chemical treatments, cells were washed with PBS and fixed using ice cold 70% EtOH o/n. The following day, the cells were washed twice with PBS and DNA was stained using FxCycle PI/RNase Staining Solution (Thermo Fisher Scientific, Cat#F10797). After 30 min staining the cells were analyzed using BD Biosciences flow cytometer LSR II HTS with excitation lasers at 561 nm and emission filter at 610/20. Estimations of ploidy levels during SMR measurements were done based on cell mass, as we noticed that cells approximately doubled their mass during every endomitotic cycle.

SMR data analysis and statistics

Small-channel SMR frequency data was analyzed using the custom MATLAB code detailed previously (2). Large-channel SMR frequency data was analyzed using similar code, which extracts first mode peaks instead. The resonant frequency changes upon cell transit were converted to buoyant mass using sensitivity factors obtained from polystyrene bead measurements. The resulting buoyant mass measurement and time data were then analyzed for growth rates (pg/h) using the custom MATLAB code published previously (1) with the following modifications:

For small-channel SMR data, mass data were linearly interpolated to have 1000 mass data points for every hour. After interpolation, data was filtered using a moving average of 100 points. Smoothed data was then fitted with a 15 min long linear line, the slope of which is the growth rate, and the fitting was moved 5 min at a time to cover the whole cell cycle. An exception to this is was when growth efficiency was analyzed in a specific cell cycle stages (Fig. 2C), as in these analyses, the line fitting was carried out on a 1 h long segments of raw data without any interpolation. Also, for the correlation of time at maximum growth efficiency with cell cycle length (Figs. S5E,F), an additional moving average filter of size 3 was imposed on mass and growth rate after obtaining the 15 min fittings, in order to minimize noise in the estimated timing of maximum growth efficiency.

For large-channel SMR data, the mass data were also interpolated to have 1000 mass data points for every hour and resulting interpolated data was filtered using a moving average of 100 points. Smoothed data was then fitted with a 60 min long linear line, the slope of which is the growth rate, and the fitting was moved 5 min at a time to cover the whole cell cycle.

We assigned the following cell cycle transitions to mass traces: G1/S transition (small-channel SMRs only) was assigned at the point where the mAG-hGeminin signal started increasing; G2/M transition was assigned based on reduced acoustic signal (small-channel SMRs only) and increased mass accumulation rate as detailed before (1, 2); metaphase/anaphase transition was assigned based on loss of mAG-hGeminin signal (small-channel SMRs only), radical drop in acoustic signal (small-channel SMRs only) and minor loss of buoyant mass as detailed before (1, 2); and cytokinesis was assigned as the point where cell mass suddenly reduced by half (abscission of daughter cells). Using these transition points, we excluded mitosis (from G2/M transition to 15 min after metaphase/anaphase transition) from our growth analyses. When analyzing growth efficiency as a function of cell mass in specific points in the cell cycle, we defined newborn G1 as the 60 min period starting 10 min after division, G1/S transition as the 60 min period surrounding the assigned G1/S point, and late G2 as the last 60 min prior to G2/M transition.

To derive the errors in growth efficiency measurements in each specific stage in the cell cycle (Fig. 2C), we utilized the standard deviation of the slope of the fitted linear line ($\delta\ Growth\ rate$) and the standard deviation of a typical mass measurement ($\delta\ mass$) which was determined previously (1). These errors were used to calculate the cumulative error (δ Growth efficiency) according to the formula:

$$\delta\ \text{Growth efficiency} = |\text{Growth efficiency}| \sqrt{\left(\frac{\delta\ Growth\ rate}{Growth\ rate}\right)^2 + \left(\frac{\delta\ mass}{mass}\right)^2}$$

For all samples, except for RO-3306 treated cells, we only analyzed mass traces that contained one or more mitosis. When cells died while trapped within the SMR, we excluded the part of the data where mass accumulation was zero or negative but included all data prior to the point where mass accumulation stopped (see *SI Appendix*, Fig. S6C for examples). When analyzing control cells using the small-channel SMR, we always monitored the cells for multiple cell cycles to verify that our analysis focused on actively growing and proliferating cells. Partial cell cycles in the control mass traces were not analyzed. No RO-3306 treated mass traces were excluded, but we stopped our data analysis after one doubling of cell mass as long-term drug treatments resulted in cell death. Errors in growth rates and growth efficiencies across the cell cycle (e.g. Figs. 2A, 3E) were plotted by overlaying the individual growth traces and plotting the mean ± standard deviation (representing both technical error and cell-to-cell variability) of the cells at each point, as previously described (1).

The quantification of cell size dependent growth was carried out using Barasertib treated L1210 cell data from the large-channel SMRs exclusively, in order to minimize influence from drug induced toxicity (between control and drug treated experiments), and from system specific biases, such as differences in shear stress, temperature, light induced toxicity and acoustic effects in the SMR channel (2). The cell size dependent growth was determined based on the slope of a linear line that was fitted to the growth efficiency data spanning five cell cycles (five size doublings) (see *SI Appendix*, Fig. S7D). To minimize influence from cell cycle dependent effects, the fitting was started and stopped at the same point in the cell cycle, by assuming an exact doubling of buoyant mass within each endomitotic cycle. The cell cycle dependent growth efficiency was determined by comparing the maximal and minimal growth efficiency observed within a cell cycle in control cells (excluding mitosis). These cell size and cell cycle dependent growth efficiencies were then compared to the average growth efficiency of control cells during whole cell cycle to derive the % to which cell size and cell cycle influence growth within a typical cell cycle. The error of this % value represents the compound error of average growth rate and that of cell cycle or size dependent growth rate (calculated similarly as the cumulative errors detailed above).

In figures displaying population average growth rates or efficiencies as a function of mass, as well as single-cell heatmaps, we only display data points (masses) that have data from three or more independent experiments. All data processing and plotting was done using MATLAB and statistical tests were carried out using MATLAB or Origin Pro 8 software.

Figures S1 to S8 with figure legends

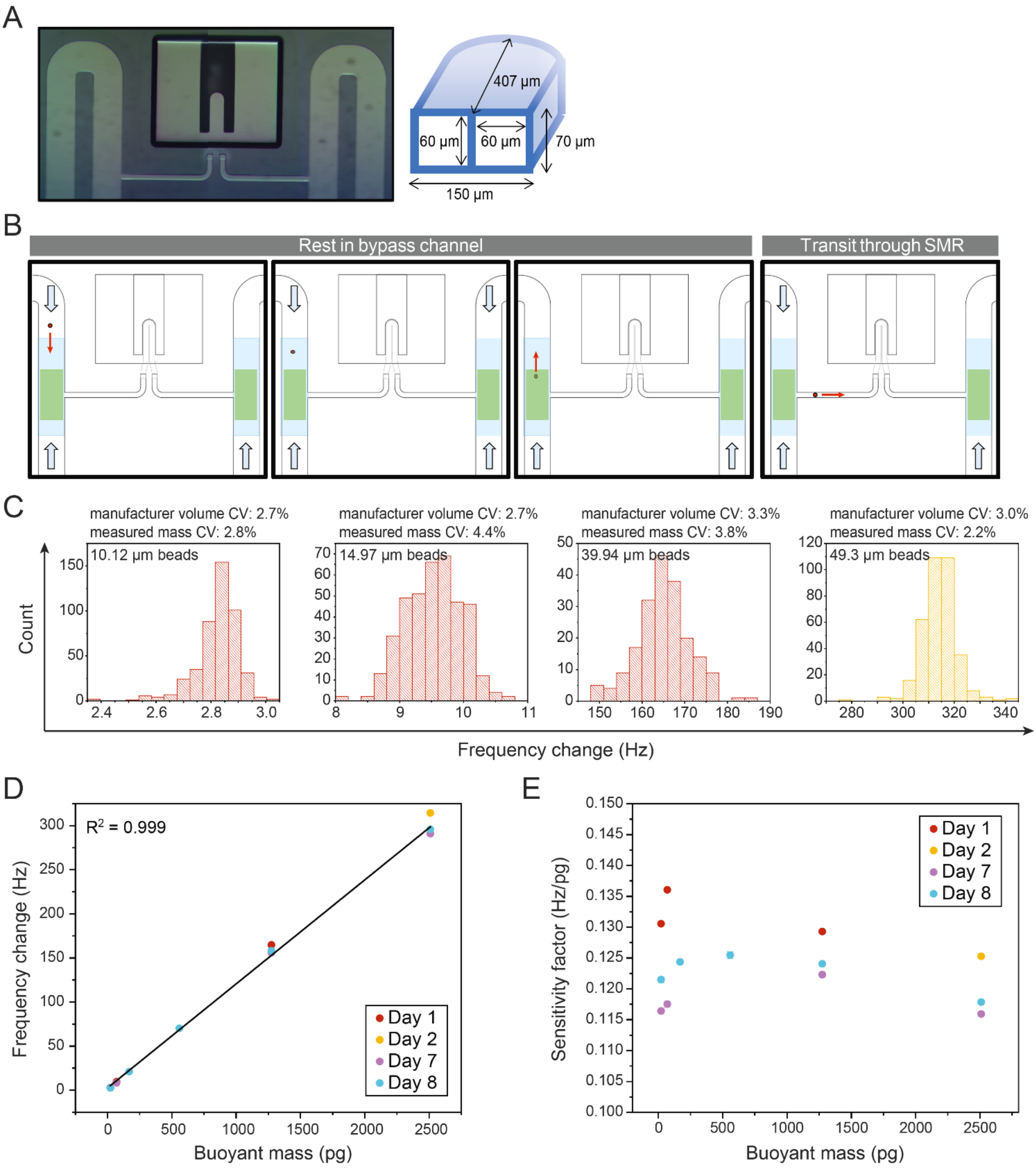

Fig. S1. Large-channel SMR employs image-based trapping and expands the measurement range of SMRs.

(A) A microscopy image (left) and an illustration showing the dimension (right) of a large-channel SMR. The image was reconstructed from three fields of views.

(B) Schematic of the image-based hydrodynamic trapping. Four regulators are used to control the pressures at the four ports through which fluid is flown in to the system. Cell (red dot) remain in the bypass channel between each successive mass measurements. During this time the image-based

hydrodynamic trapping maintains the cell inside the blue shaded region of interest (ROI), but outside the green shaded ROI using the following strategy. If the cell flows out of the ROIs (1st panel), the pressures (large light blue arrows) on four ports are set such that the cell flows towards the SMR cantilever in the middle (direction of cell movement is shown with thin red arrow). If the cell is inside the blue ROI, but outside the green ROI (2nd panel), then pressures are applied on all four ports to minimize cell movement. If the cell enters the green ROI (3rd panel), a minor backwards flow is triggered to prevent the cell from accidentally entering the SMR cantilever. After a defined rest period (typically 30 – 60s), the pressures are set so that the cell flows through the SMR cantilever to the other side bypass channel (right side pane), where the imaging-based hydrodynamic trapping is repeated.

(C) Example histograms of 10.12 µm (n = 459 beads), 14.97 µm (n = 394 beads), 39.94 µm (n = 196 beads), and 49.3 µm (n = 351 beads) diameter polystyrene bead measurements using the large-channel SMR. Both the measured coefficient of variability (CV) in mass and the manufacturer's reported CV in volume are shown. Data is colored by the date of measurement (same data as Day 1 and Day 2 shown in panels D & E).

(D) Large-channel SMR sensitivity scaling obtained by measuring polystyrene beads of different sizes over multiple days. Linear fit (black line) and Pearson correlation (R^2) are also shown. Data represents median (points) ± s.e.m. frequency change of the measured distribution. Note that the error is smaller than the size of the points. Buoyant mass is calculated based on bead diameter (provided by manufacturer), assuming spherical shape and fixed polystyrene density. Data is colored by the date of measurement. Device was thoroughly flushed with PBS between successive experiments on the same day and was further cleaned and passivated between measurements on different days (see *SI Appendix Large Channel SMR Operation*).

(E) Large-channel SMR sensitivity factor as a function of polystyrene bead mass, as measured over multiple days (same data as in panel D). Data represents median (points) ± s.e.m. frequency change of the measured distribution. See *SI Appendix: Large Channel SMR Measurement Error and Linearity* for more details.

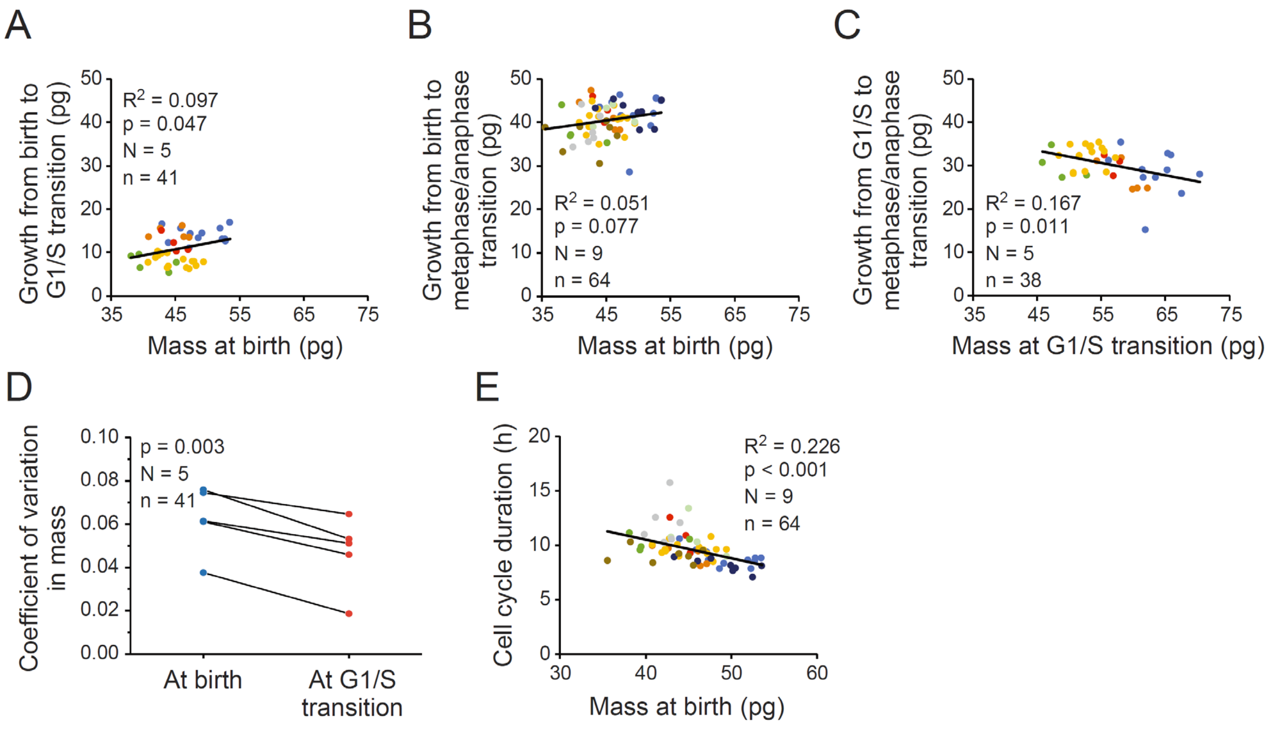

Fig. S2. Cell size control mechanism in L1210 FUCCI cells.
(A) Correlation between L1210 FUCCI cell mass at birth and the amount of growth from birth to G1/S transition.
(B) Correlation between L1210 FUCCI cell mass at birth and the amount of growth from birth to metaphase/anaphase transition (mitosis).
(C) Correlation between L1210 FUCCI cell mass at G1/S transition and the amount of growth from G1/S transition to metaphase/anaphase transition (mitosis).
(D) Cell mass variability of L1210 FUCCI cells at birth and at G1/S transition for each independent experiment (two dots connected with a line represents a single cell lineage). p-value was calculated using Student's paired two-tailed t-test.
(E) Correlation between L1210 FUCCI cell mass at birth and the duration of cell cycle (from birth to metaphase/anaphase transition).

In panels (A-C) and (E), data from each independent experiment (N) is represented in its own color and each dot represents a single cell (n). Linear fits, Pearson correlations (R^2) and p-values for the correlations (2-tailed test of significance) are shown in black.

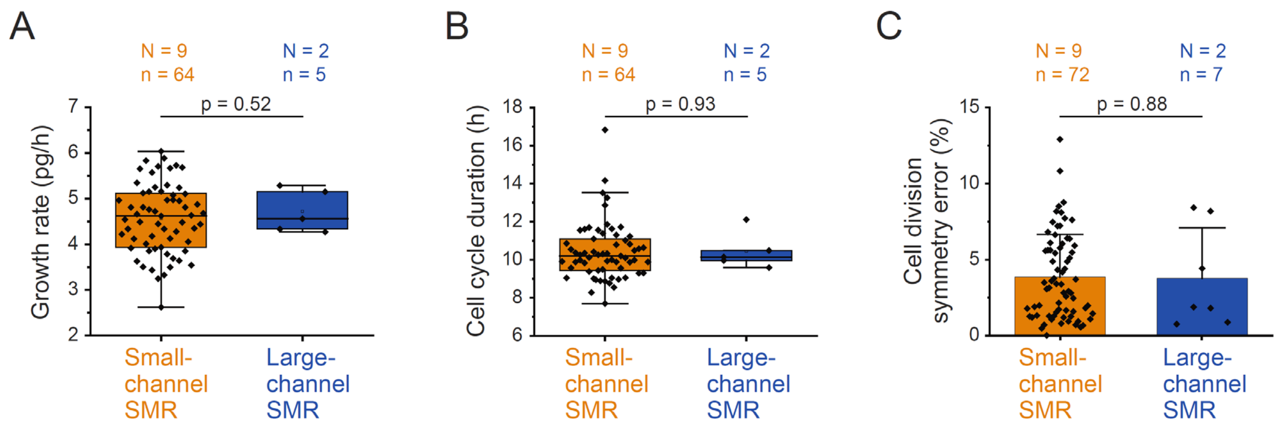

Fig. S3. Cell growth comparison between small and large-channel SMRs.
(A) L1210 FUCCI cell growth rate over a whole cell cycle in small and large-channel SMRs. p-value was obtained using Welch's t-test.
(B) L1210 FUCCI cell cycle duration in small and large-channel SMRs. p-value was obtained using Welch's t-test.
(C) L1210 FUCCI cell division symmetry in small and large-channel SMR. p-value was obtained using Mann-Whitney U test.

In panels (A-C), N and n represents independent experiments and number of single cells, respectively. In panels (A) and (B), boxes denote interquartile range and whiskers indicate 1.5 times the interquartile range. In panel (C), bars represent the mean and error bars represent S.D.

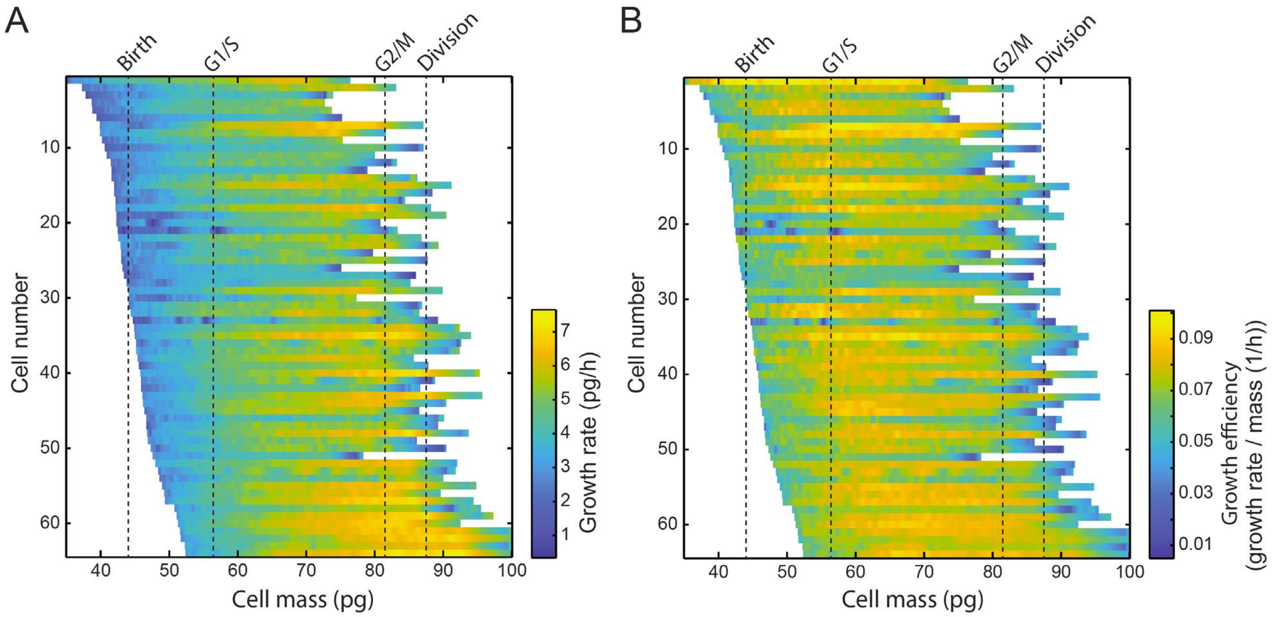

Fig. S4. Non-linear growth behavior during the cell cycle is observable in individual cells. (A,B) Heatmap of L1210 FUCCI cell growth rates (A) and growth efficiencies (B) in unperturbed (freely proliferating) cells. For both panels, each line represents a separate cell cycle (N=9 independent experiments, n=64 cells). Average newborn size (Birth), G1/S transition size, mitotic entry size (G2/M) and division size are indicated with dashed vertical lines. Growth scales non-linearly with size during each cell cycle in all individual cells.

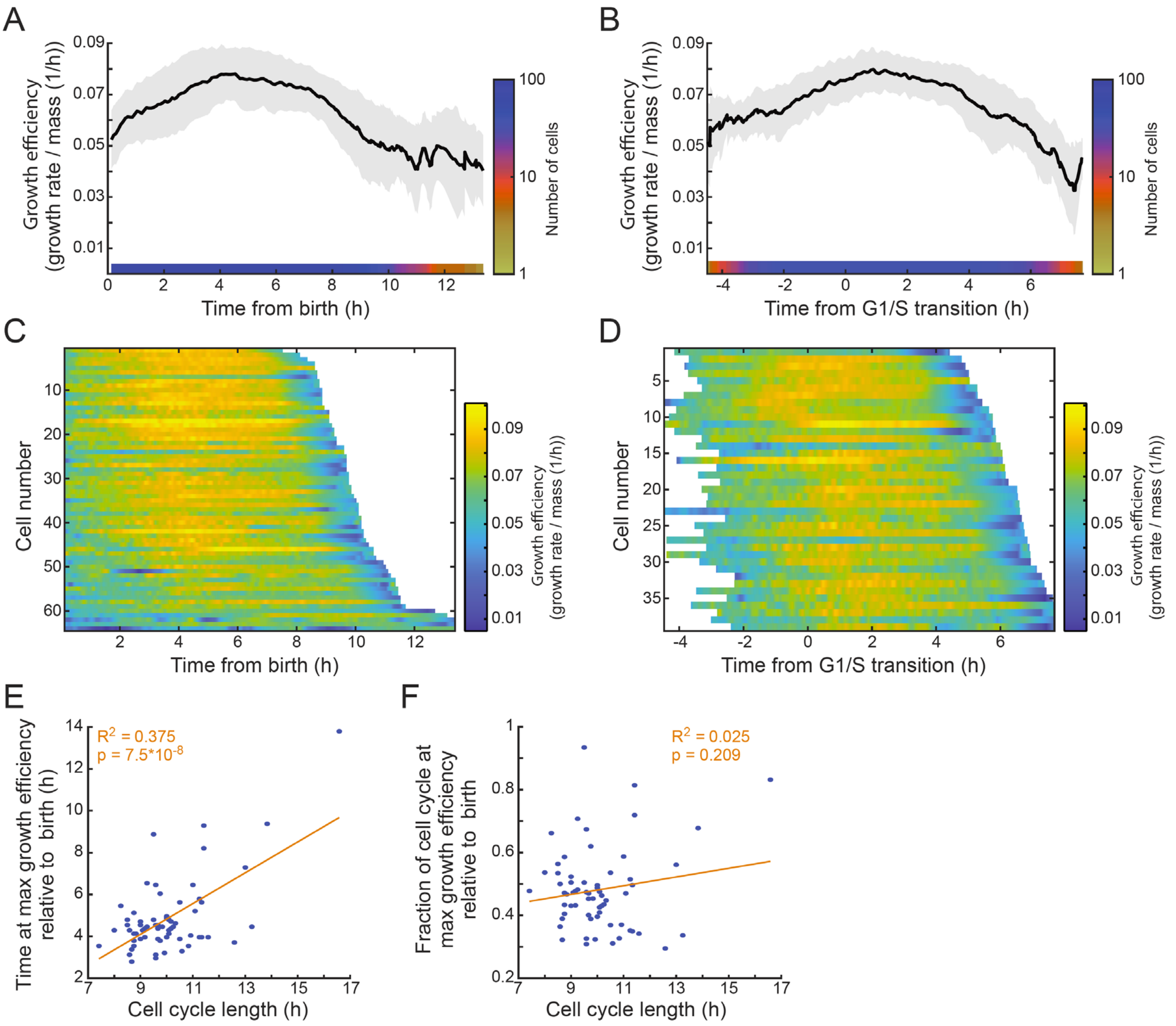

Fig. S5. Correlating growth efficiency with cell cycle time

(A,B) The growth efficiency of L1210 FUCCI cells as a function of time from birth (A) and time from G1/S transition (B) obtained using small-channel SMR. The line and shaded area indicate mean ± S.D. Number of cells for each timepoint analyzed is indicated with a color gradient at the bottom (N=9 independent experiments, n=64 cells for panel A; N=5 independent experiments, n=39 cells for panel B).

(C,D) Heatmaps of L1210 FUCCI cell growth efficiency as a function of time from birth (C) and time from G1/S transition (D). For both panels, each line represents a separate cell cycle (N=9 independent experiments, n=64 cells for panel C; N=5 independent experiments, n=39 cells for panel D).

(E, F) Scatter plots of time since birth (E) and fraction of cell cycle (F) when growth efficiency is maximized as a function of total cell cycle length (N=9 independent experiments, n=64 cells). Each dot represents a single cell. If maximum growth efficiency is achieved at a fixed amount of time since birth, the timing of maximum growth efficiency would be independent of the total cell cycle length (E). Since a positive correlation exists in (E), but the correlation becomes insignificant in (F), this suggests that the maximization of growth is linked to cell cycle. Linear fits, Pearson correlations (R^2) and p-values for the correlations (2-tailed test of significance) are shown in orange.

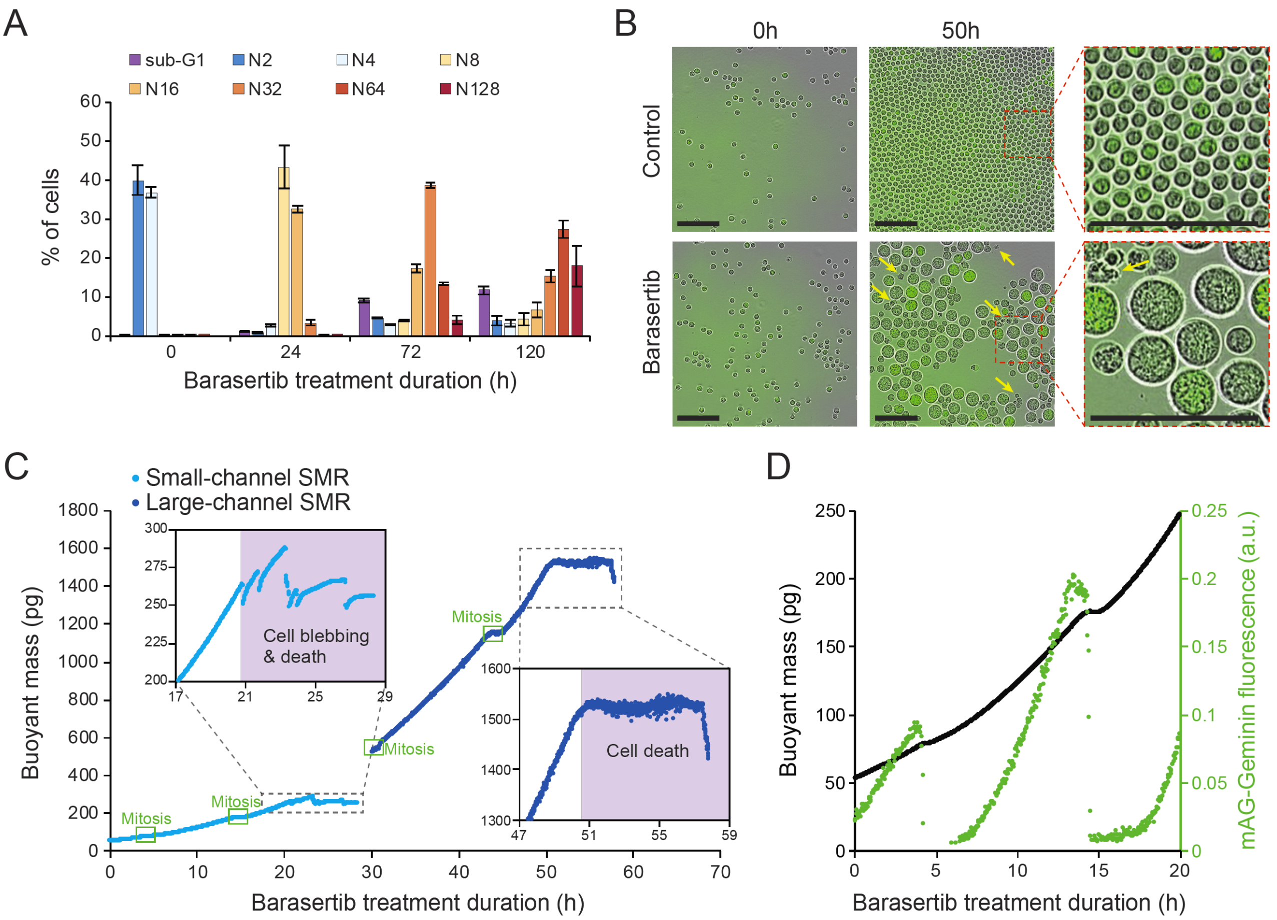

Fig. S6. Barasertib treatment results in polyploidy, cellular hypertrophy and abrupt cell deaths.

(A) Quantification of DNA content (ploidy level) in L1210 FUCCI cells following different duration 50 nM Barasertib treatments (n=3 independent cultures). Note that while cells become polyploid up to 128N, with increasing treatment times also the sub-G1 population increases, indicative of apoptotic particles. Data represents mean ± S.D.

(B) Representative phase contrast (grey) and mAG-hGeminin cell cycle reporter (green) overlaid images of L1210 FUCCI cells following 50 nM Barasertib treatment (N=2 independent experiments, n=6 independent cultures). Yellow arrows indicate examples of dead/dying cells. Also note the round cell morphology despite a radical volume increase. Scale bars denote 100 µm.

(C) Two example buoyant mass traces of 50 nM Barasertib treated L1210 FUCCI cells obtained using small (light blue) and large-channel (dark blue) SMRs. In these two examples cell growth suddenly stopped, and cells experienced cell blebbing and/or cell death. Once cell growth stopped (light purple shaded areas in the zoom-ins), data was excluded from further analysis. Mitotic events are marked in green and should not be confused with cell death.

(D) Example buoyant mass (black) and mAG-hGeminin cell cycle reporter (green) trace of a 50 nM Barasertib treated L1210 FUCCI cells obtained using small-channel SMR. The loss of the mAG-hGeminin signal indicates metaphase-anaphase transition in mitosis and the increase in mAG-hGeminin indicates G1/S transition, indicating that the cell is undergoing endomitotic cycles.

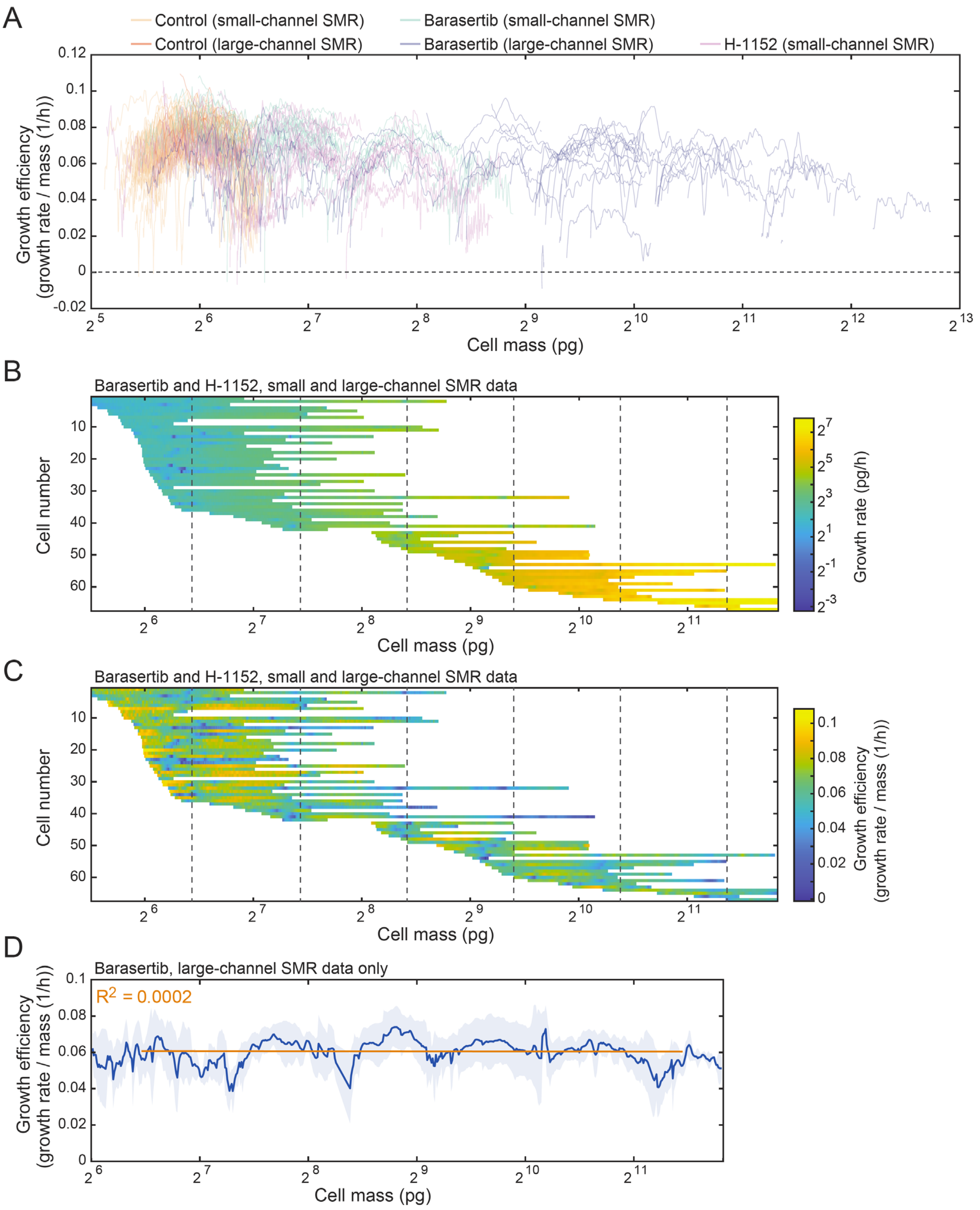

Fig. S7. Each endomitotic cycle displays non-linear growth efficiency scaling, but growth remains exponential over large size scales

(A) The growth efficiency of control (N=9 independent experiments, n=64 cells for small-channel SMR; N=2 independent experiments, n=9 cells for large-channel SMR), 50 nM Barasertib (N=19 independent experiments for small-channel SMR; N=31 independent experiments for large-channel SMR) and 10 μM H-1152 (N=17 independent experiments for small-channel SMR) treated L1210 FUCCI cells. Each cell is indicated as an opaque line with gaps between lines representing mitosis, which were not used in the analysis.

(B, C) Heatmap of L1210 FUCCI cell growth rates (B) and growth efficiencies (C) following 50 nM Barasertib or 10 µM H-1152 treatments. For both panels, each heatmap line represents a separate experiment (see panel (A) for number of independent experiments). Dashed vertical lines indicate typical mitotic sizes. Growth rates of all cells increase with size during polyploidization (B). However, in each endomitotic cycle, cells display non-linear scaling of growth efficiency (C).
(D) Growth efficiency of Barasertib treated L1210 FUCCI cells measured using large-channel SMR only (N=31 independent experiments). The line and shaded area indicate mean ± S.D. The size-dependency of growth was analyzed using this dataset in order to avoid comparisons across multiple conditions (e.g. chemical treated vs untreated). Cell size dependency of growth efficiency was analyzed based on a linear fit (orange line) to a range covering 5 cell size doublings. Pearson correlation (R^2) of the fit is also displayed.

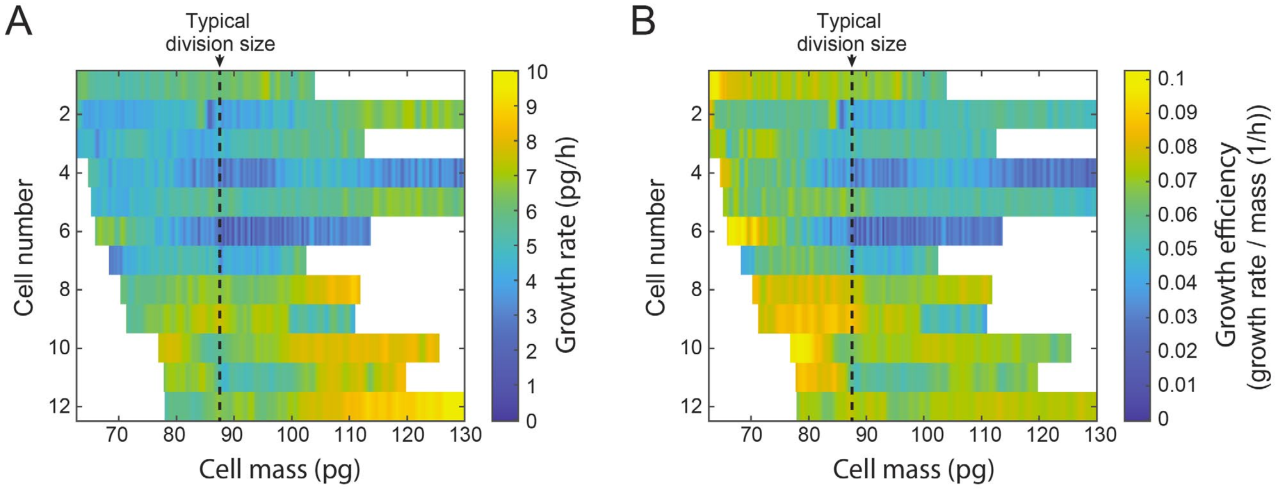

Fig. S8. Single-cell growth behavior following RO-3306 mediated G2 arrest.
(A,B) Heatmap of L1210 cell growth rates (A) and growth efficiencies (B) following 2 μM RO-3306 treatment. For both panels, each line represents a separate cell and experiment (N=12 independent experiments, n=12 cells). Dashed vertical line indicates the typical division size of control cells. As the G2 arrested cells grow larger than the normal division size, growth rates keep increasing and efficiency remains constant for most cells.

Dataset S1 (separate file).

This excel file contains datasets used to analyze cell size and cell cycle-dependent growth in the manuscript by Mu, Kang, et al. The first data sheet contains information regarding the timing of cell cycle transitions in the control L1210 FUCCI cells analyzed using small-channel SMR. The rest of the data sheets contain time (from the start of the trace, not from the start of the drug treatment) and buoyant mass measurement data. Each sheet contains all the data for that specific experiment type, with cell type, drug treatment and measurement device detailed in the sheet name.

SI References

1. T. P. Miettinen, J. H. Kang, L. F. Yang, S. R. Manalis, Mammalian cell growth dynamics in mitosis. *Elife* **8** (2019).
2. J. H. Kang *et al.*, Noninvasive monitoring of single-cell mechanics by acoustic scattering. *Nat Methods* **16**, 263-269 (2019).
3. S. Son *et al.*, Direct observation of mammalian cell growth and size regulation. *Nat Methods* **9**, 910-912 (2012).
4. J. H. Kang *et al.*, Time-dynamics of mitochondrial membrane potential reveal an inhibition of ATP synthesis in mitosis. *BioRxiv* 10.1101/772244 (*2019*).
5. N. Cermak *et al.*, High-throughput measurement of single-cell growth rates using serial microfluidic mass sensor arrays. *Nat Biotechnol* **34**, 1052-1059 (2016).
6. S. Olcum, N. Cermak, S. C. Wasserman, S. R. Manalis, High-speed multiple-mode mass-sensing resolves dynamic nanoscale mass distributions. *Nat Commun* **6**, 7070 (2015).
7. T. P. Miettinen, M. Bjorklund, Mevalonate Pathway Regulates Cell Size Homeostasis and Proteostasis through Autophagy. *Cell Rep* **13**, 2610-2620 (2015).
